# The Preparation of Dithieno[3,2-*b*:4,5-*c*’]germole, and Its Application as a Donor Unit in Conjugated D–A Compounds

**DOI:** 10.3390/molecules29153553

**Published:** 2024-07-28

**Authors:** Cong-Huan Wang, Yohei Adachi, Joji Ohshita

**Affiliations:** 1 Smart Innovation Program, Graduate School of Advanced Science and Engineering, Hiroshima University, Higashi-Hiroshima 739-8527, Japan; d210833@hiroshima-u.ac.jp (C.-H.W.); yadachi@hiroshima-u.ac.jp (Y.A.); 2 Division of Materials Model-Based Research, Digital Monozukuri (Manufacturing) Education and Research Center, Hiroshima University, Higashi-Hiroshima 739-0046, Japan

**Keywords:** germole, donor unit, donor–acceptor compound, conjugated materials

## Abstract

Group 14 metalloles have attracted much attention as core structures of conjugated functional materials. In this work, we prepared dithieno[3,2-*b*:4,5-*c*’]germole as a new unsymmetrically condensed dithienogermole and benzo[4,5]thieno[2,3-c]germole as the benzene-condensed analog. The electronic states and properties of these unsymmetrically condensed germoles are discussed on the basis of the results of optical and electrochemical measurements with the help of quantum chemistry calculations on the simplified model compounds. The Stille cross-coupling reactions of bromodithieno[3,2-*b*:4,5-*c*’]germole with di(stannylthienyl)- and di(stannylthiazolyl)benzothiadiazole provided conjugated donor–acceptor compounds that exhibited clear solvatochromic behavior in the photoluminescence spectra, indicating the potential application of the dithieno[3,2-*b*:4,5-*c*’]germole unit as an electron donor in donor–acceptor systems.

## 1. Introduction

**Figure 1:**
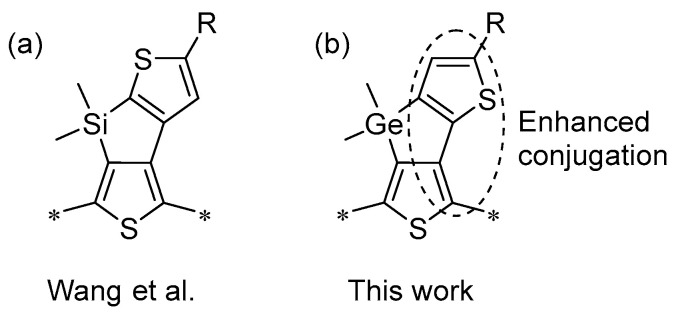
Structures of dithieno[2,3-b:4,5-c’]silole [8,9] (**a**) and dithieno[3,2-b:4,5-c’]germole (**b**) where asterisks indicate potential junctions to conjugated systems.

Functional organic π-conjugated molecules have garnered significant attention because of their wide-ranging applications, including their use in optoelectronic devices. Group 14 metalloles have been extensively studied as core structures of conjugated functional materials [1]. For example, conjugated compounds and polymers containing silole units, such as poly(silole)s, are anticipated to exhibit effective conjugation, making them suitable for both n- and p-type semiconductor materials depending on the substituent [2]. This is explained by the low-lying LUMO energy levels due to σ*-π* orbital interactions and the high planarity of the silole system. Dithienosilole is a typical silole-containing building block that is used for the development of conjugated organic materials [3,4,5,6]. However, most research activities have focused on symmetrically condensed dithienosiloles, and less is known for unsymmetrical dithienosiloles [7,8,9]. H. Wang and coworkers have prepared dithieno[2,3-b:4,5-c’]silole derivatives and produced conjugated polymers using them as the monomers (Figure 1a) [8,9]. In this unsymmetrical unit, one thiophene ring can be incorporated into conjugated compounds and polymeric systems, and the introduction of a functional group (R) on the other thiophene ring enables the fine-tuning of the electronic state of the dithienosilole unit. Meanwhile, within the same group as silicon, germanium-based congeners known as dithienogermoles are relatively underexplored despite exhibiting properties that are comparable to or even superior to those of their Si analogs, arising from their relatively strong intermolecular interactions [10,11,12,13]. Furthermore, dithienogermoles generally demonstrate a higher stability than dithienosiloles [14]. Therefore, the exploration of unsymmetrical dithienogermoles as a promising building block for the development of functional organic optoelectronic device materials would facilitate the efficient construction of germole-containing conjugated compounds and polymers and have significant implications.

In the course of our studies on unsymmetrically condensed metalloles [15,16], we prepared dithieno[3,2-b:4,5-c’]germole (uDTG) as the first example of group 14 dithieno[3,2-b:4,5-c’]metalloles. We anticipate that uDTG would show enhanced conjugation compared with the dithieno[2,3-b:4,5-c’]siloles (Figure 1), thereby paving the way for the development of building units of conjugated functional materials with finely tuned electronic states. We also prepared benzo[4,5]thieno[2,3-c]germole (BTG). The optical and electrochemical properties of these thiophene-condensed unsymmetrical germole derivatives were investigated and discussed with the help of quantum chemical calculations. To explore the usefulness of uDTG as the building unit of conjugated compounds and polymers, we synthesized two donor–acceptor (D–A) compounds possessing uDTG as the donor and benzo(2,1,3)thiadiazole (BT) as the acceptor, which were linked by thiophene or thiazole units. These compounds showed clear solvatochromic behavior arising from their intramolecular D–A interaction, and their photoluminescence (PL) bands shifted to lower energies as the solvent polarity increased. The compounds described herein are expected to shed light on the design and application of new building blocks of conjugated compounds and polymers.

## 2. Results and Discussion

### 2.1. Synthesis

**Scheme 1:**
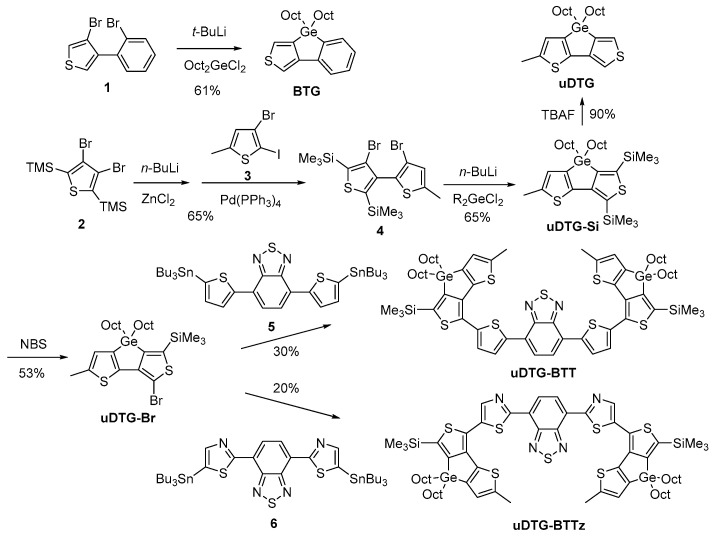
Synthesis of unsymmetrically condensed thienogermoles.

The synthetic strategy for unsymmetrically condensed benzothieno- and dithienogermoles is presented in Scheme 1. Compound **1** was prepared according to the previously reported method [17] and used for the synthesis of **BTG**. For the formation of **uDTG** derivatives, compound **4** was first obtained by a Negishi cross-coupling reaction of compounds **2** and **3** in 65% yield [18,19]. The dilithiation of **4** by *n*-BuLi, followed by a ring-forming reaction with dichlorodi(*n*-octyl)germane furnished a mixture from which **uDTG-Si** was isolated in 65% yield by preparative gel permeation chromatography (GPC). **uDTG-Si** was then treated with tetrabutylammonium fluoride (TBAF) to give desilylation product **uDTG** in 90% yield. The bromination of **uDTG-Si** with NBS gave **uDTG-Br** in 53% yield. This compound was further subjected to palladium-catalyzed Stille coupling with **5** and **6** to provide **uDTG-BTT** and **uDTG-BTTz** as dark red solids in 30% and 20% yields, respectively. The low yields of these compounds were due to the low reactivity of the starting materials and difficulty in purification. We found that the starting materials remained in the reaction mixtures by NMR spectral analysis.

### 2.2. Optical and Electrochemical Measurements

**Figure 2:**
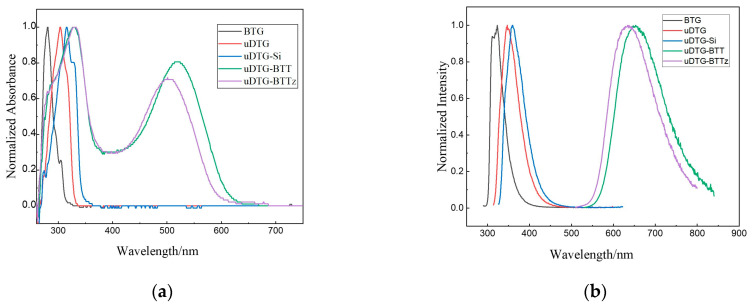
UV–vis absorption spectra (**a**) and PL spectra (**b**) of unsymmetrically condensed thienogermoles in dichloromethane (1.0 × 10^−5^ M) at room temperature.

**Figure 3:**
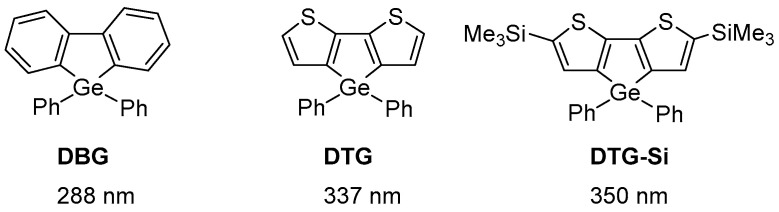
UV–vis absorption maxima for symmetrically condensed germoles in the literature.

We examined the optical and electrochemical properties of the prepared **BTG** and uDTS derivatives to clarify their electronic states. The UV–vis absorption spectra of **BTG**, **uDTG**, **uDTG-Si**, **uDTG-BTT**, and **uDTG-BTTz** in dichloromethane are shown in Figure 2a, and the corresponding photophysical data are summarized in Table 1. As shown in Figure 2a, **BTG** displayed an absorption maximum at 280 nm. Upon replacing the condensed benzene ring of **BTG** with a thiophene ring, an absorption maximum appeared at 303 nm with a bathochromic shift of 23 nm for **uDTG**. A further bathochromic shift was observed for **uDTG-Si**, giving an absorption maximum at 315 nm. Their PL spectra showed similar bathochromic shifts, as presented in Figure 2b and Table 1. This bathochromic shift from **BDG** to **uDTG** is in agreement with that observed for symmetrically condensed dibenzo and dithienosiloles (**DBG** and **DTG** in Figure 3) [20,21]. The absorption band of **uDTG-Si** appeared at lower energy than that of **uDTG** similar to a previous report on DTG derivatives that showed a bathochromic shift of the absorption band by similar silyl substitution (**DTG-Si** in Figure 3) [22]. Hypsochromic shifts of the absorption bands of uDTG derivatives relative to those of the symmetrically condensed DTG analogs [20,21,22] are likely due to the less extended π-conjugation of the α,β-linked bithiophene units in **uDTG** and **uDTG-Si** compared with the α,α-linked bithiophene units in **DTG** and **DTG-Si**.

D–A interaction was noted in **uDTG-BTT** and **uDTG-BTTz**, which are composed of uDTG and BT units as the donor and acceptor units, respectively, linked by thiophene or thiazole units. The UV–vis absorption spectra of these compounds showed two π–π* transition bands around 330 nm and 500 nm. The former is ascribable to the local excitation, whereas the latter is likely based on the D–A interaction that leads to photoexcited intramolecular charge transfer (ICT). The absorption maxima in the low-energy region were observed at 520 nm and 501 nm for **uDTG-BTT** and **uDTG-BTTz**, respectively. The blue-shifted absorption maximum of **uDTG-BTTz** relative to that of **uDTG-BTT** is explained by the less extended conjugation of imine-containing thiazole than thiophene [23,24]. The more electron-deficient property of thiazole may also affect the donor–acceptor interaction of these compounds. The optical band gaps of the unsymmetrically condensed thienogermoles were estimated from the absorption edges, decreasing in the order of **BTG** > **uDTG** > **uDTG-Si** > **uDTG-BTTz** > **uDTG-BTT**, as shown in Table 1.

**Figure 4:**
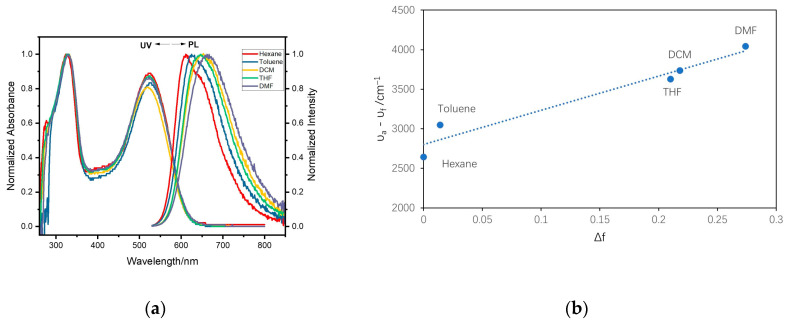
UV absorption (solid line) and PL spectra (dotted line) in different solvents (**a**), and the Lippert–Mataga plot (**b**) of **uDTG-BTT**. The fitted line with a slope of 4266 cm^−1^ (R^2^ = 0.95) is shown.

Monomeric compounds **BTG**, **uDTG**, and **uDTG-Si** showed PL bands around 323–360 nm with low PL efficiencies. In contrast, compounds **uDTG-BTT** and **uDTG-BTTz** exhibited strong PL bands at 653 nm and 634 nm, respectively. Many donor–acceptor compounds with a benzothiadiazole donor unit with red-shifted absorption and PL bands with sufficient PL efficiencies for optical applications have been reported [23,24,25]. To understand the ICT behaviors of **uDTG-BTTz** and **uDTG-BTT**, the PL spectra were measured in different solvents, and the results are shown in Figure 4, Figures S1 and S2 and Table S1. The PL band shifted to the longer wavelength region with increasing solvent polarity, clearly indicating that the photoexcited states of **uDTG-BTT** and **uDTG-BTTz** were more polar than their respective ground states, reflecting the ICT behavior. From the slope of the Lippert–Mataga plot, the dipole moment changes (Δμ) were estimated to be 13.03 D and 15.57 D for **uDTG-BTT** and **uDTG-BTTz**, respectively.

The HOMO and LUMO energy levels of conjugated materials are important parameters for electronic applications. Cyclic voltammogram (CV) measurements were performed in a typical three-electrode electrochemical cell with Pt as the working electrode in water-free acetonitrile at a scan rate of 50 mV s^−1^ (Figure S3) to determine the energy levels. The HOMO energy levels determined from the onsets of the first oxidation peaks are −6.27 eV for **BTG**, −5.50 eV for **uDTG**, and −5.52 eV for **uDTG-Si**. **uDTG-BTTz** possessed a lower HOMO energy level than **uDTG-BTT** owing to the more electron-deficient nature of thiazole than thiophene [23,24]. The LUMO energy levels of the compounds were calculated from the anodic onsets and the optical band gaps and are listed in Table 1. The HOMO and LUMO of **uDTG-BTT** are at –5.08 eV and −3.04 eV, whereas those of **uDTG-BTTz** are at −5.16 eV and −3.05 eV, providing similar bandgaps of 2.04 and 2.11 eV, respectively.

### 2.3. Quantum Chemical Calculations

**Figure 5:**
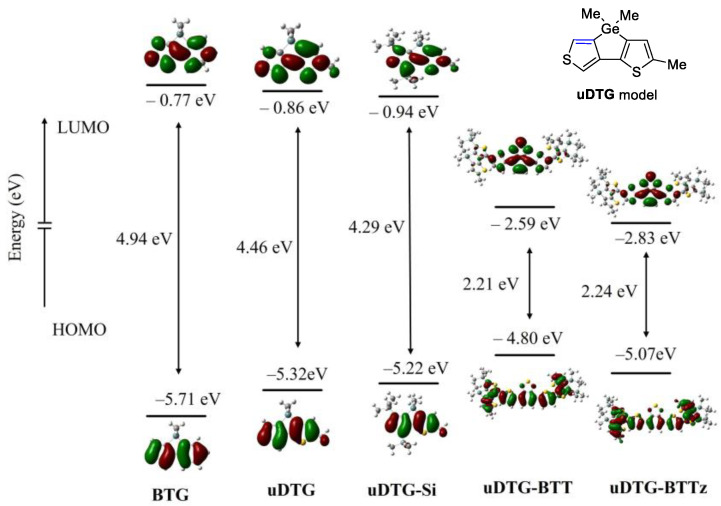
Energy levels and profiles of **BTG**, **uDTG**, **uDTG-Si**, **uDTG-BTT**, and **uDTG-BTTz** derived from DFT calculation at the B3LYP/6-31G(d,p) level of theory in the gas phase, with alkyl chains replaced by methyl groups to simplify the calculations. The structure of **uDTG** model is also seen with the vinylene unit at the C5 position of germole in blue.

To obtain further insight into the effects of molecular structure and electron distribution on the spectroscopic and electrochemical properties of these compounds, their electronic structures were investigated with the Gaussian 16 suite of quantum chemical simulation programs. The structures were optimized in vacuo using DFT calculations at the B3LYP/6-31G(d,p) level of theory. The visualized HOMO and LUMO distributions and the calculated energy levels are shown in Figure 5. During the calculations, the compounds were optimized, where octyl chains on the germanium atom were replaced with methyl groups to simplify the calculations. The orbital levels of the small molecules were in good agreement with the experimental data, except that calculations predicted a higher HOMO energy level for **uDTG-Si** than for **uDTG**. This differs from the experimental data that **uDTG-Si** showed the anodic onset at a higher potential than **uDTG**, although the difference was not large. It is also seen that the vinylene unit at the C5 position of germole contributes less to the HOMO and LUMO of uDTG system than other C=C units, reflecting the unsymmetrical structure, thereby leading to less extended conjugation compared with symmetrical DTG. The LUMO/HOMO energy levels of uDTG-containing D–A compounds **uDTG-BTT** and **uDTG-BTTz** were estimated to be −2.59/−4.80 and −2.83/−5.07, respectively. Both the HOMO and LUMO of **uDTG-BTT** lie at higher levels than those of **uDTG-BTTz**, agreeing with the experimental data, reflecting the more electron-deficient property of thiazole. The LUMOs of these D–A compounds are mainly found in the acceptor BT units, whereas the HOMOs are distributed in the uDTG fragments, as shown in Figure 5. This result indicates that ICT occurs from the uDTG donor to the BT acceptor upon photoexcitation. It is interesting to see that the thiophene and thiazole linkers also affect the HOMO and LUMO energy levels to make their fine-tuning possible.

## 3. Experimental Section

### 3.1. General Preparation

All synthetic reactions were performed in an atmosphere of dry argon. Tetrahydrofuran (THF) and diethyl ether that were used as reaction solvents were dried by stirring with calcium hydride overnight at room temperature. These solvents were then distilled and stored with activated molecular sieves in the dark. Compounds **1**, **2**, **3**, **5**, and **6** were prepared by the literature procedures [17,18,19,26,27]. All other chemicals employed were commercially available and used as received. All spectral and electrochemical measurements were performed at room temperature. The spectrometer models used for the measurements are as follows: NMR spectra: Varian 400-MR; UV–vis absorption spectra: Hitachi U-2910; PL spectra: HORIBA FluoroMax-4; APCI-mass spectra: Thermo Fisher Scientific LTQ Orbitrap XL. Cyclic voltammograms (CVs) were obtained on an Autolab analyzer using a typical three-electrode electrochemical cell in an acetonitrile solution containing 0.1 M of tetrabutylammonium hexafluorophosphate as a supporting electrolyte at a scan rate of 50 mV s^−1^ at room temperature in a nitrogen atmosphere with platinum disk and wire as the working counter electrode, respectively. An Ag/Ag^+^ electrode was used as the reference electrode, calibrated by ferrocene.

### 3.2. Procedures for Synthesis

#### 3.2.1. Synthesis of **BTG**

To a solution of **1** (0.416 g, 1.308 mmol) in 10 mL of diethyl ether was added 3.27 mL (5.232 mmol) of a pentane solution of *t*-BuLi (1.6 M) at −78 °C. After the solution was stirred at this temperature for 2 h, 0.508 g (1.373 mmol) of dichlorodi(*n*-octyl)germane in 5 mL of ether was added by dropping. The reaction mixture was stirred at room temperature overnight. After being quenched with H_2_O (20 mL), the reaction mixture was extracted with CHCl_3_ (3 × 20 mL), and the extract was dried over anhydrous magnesium sulfate. After evaporation of the solvent, the residue was subjected to preparative GPC eluting with toluene to give 0.388 g (0.800 mmol, 61% yield) of **BTG** as a colorless oil. ^1^H NMR (400 MHz, CDCl_3_) δ 7.68 (d, *J* = 8.0 Hz, 1H), 7.53 (d, *J* = 7.2 Hz, 1H), 7.48 (d, *J* = 2.2 Hz, 1H), 7.37–7.32 (m, 1H), 7.33 (d, *J* = 2.2 Hz, 1H), 7.22–7.17 (m, 1H), 1.47–1.43 (m, 4H), 1.28–1.19 (m, 24H), 0.86 (t, *J* = 7.2 Hz, 6H). ^13^C NMR (100 MHz, CDCl_3_) δ 152.8, 144.8, 143.2, 141.4, 134.0, 129.3, 128.6, 126.7, 122.3, 115.0, 33.1, 32.0, 29.4, 29.3, 25.5, 22.8, 14.9, 14.3. HR-MS (APCI) Calcd for C_26_H_41_Ges: [M + H]^+^: 459.21353, Found: 459.21353.

#### 3.2.2. Synthesis of **4**

To a solution of **2** (0.775 g, 2.006 mmol) in 20 mL of THF was added 1.25 mL (2.006 mmol) of a 1.6 M hexane solution of *n*-BuLi at −78 °C, and the reaction mixture was stirred at this temperature for 1 h. Zinc chloride (2.4 mL, 2.407 mmol, 1 M in THF) was added, and the resulting mixture was further stirred at −78 °C for 1 h. Then, the reaction mixture was warmed to room temperature. After stirring for 1 h, **3** (0.607 g, 2.006 mmol) and Pd(PPh_3_)_4_ (0.116 g, 0.1003 mmol) were added and the reaction mixture was heated at 80 °C overnight. The solvent was evaporated under reduced pressure, and the residue was directly subjected to column chromatography on silica gel eluting with hexane for purification to give **4** in 65% yield (0.629 g, 1.304 mmol) as a white solid. mp 89.6–90.8 °C. ^1^H NMR (400 MHz, CDCl_3_) δ 6.73 (s, 1H), 2.50 (s, 3H), 0.42 (s, 9H), 0.17 (s, 9H). ^13^C NMR (100 MHz, CDCl_3_) δ 146.77, 142.49, 141.28, 140.88, 139.60, 128.13, 122.70, 111.91, 15.67, −0.10, −0.68. HR-MS (APCI) Calcd for C_15_H_22_Br_2_S_2_Si_2_: [M]^+^: 479.90682, Found: 479.90671.

#### 3.2.3. Synthesis of **uDTG-Si**

To a solution of **4** (0.500 g, 1.036 mmol) in 20 mL of THF was added a 1.6 M hexane solution of *n*-BuLi (1.328 mL, 2.124 mmol) at −78 °C, and the reaction mixture was stirred at this temperature for 2 h. Dichlorodi(*n*-octyl)germane (0.421 g, 1.139 mmol) was added to the reaction mixture at −78 °C. After stirring overnight at room temperature, the solvent was evaporated under reduced pressure, and the residue was directly purified by preparative GPC eluting with toluene to give **uDTG-Si** in 65% yield (0.249 g, 0.673 mmol) as a colorless oil. ^1^H NMR (400 MHz, CDCl_3_) δ 6.71 (s, 1H), 2.54 (s, 3H), 1.41–1.33 (m, 4H), 1.25–1.18 (m, 24H), 0.86 (t, *J* = 7.2 Hz, 6H), 0.48 (s, 9H), 0.33 (s, 9H). ^13^C NMR (100 MHz, CDCl_3_) δ 154.77, 154.56, 147.72, 147.06, 146.09, 140.98, 131.40, 128.02, 33.08, 31.97, 29.37, 29.23, 25.76, 22.81, 16.16, 15.59, 14.25, 0.81, 0.35. HR-MS (APCI) Calcd for C_31_H_57_GeS_2_Si_2_: [M + H]^+^: 623.26465, Found: 623.26624.

#### 3.2.4. Synthesis of **uDTG**

To a solution of **uDTG-Si** (0.399 g, 0.642 mmol) in THF (15 mL) was added a solution of TBAF (0.642 mmol, 1 M) in THF at 0 °C, and the reaction mixture was stirred for 10 min at this temperature. After evaporation of the solvent, the residue was diluted with 10 mL of ether acetate and hydrolyzed with 3 × 10 mL water. The organic layer and the extracts were combined and dried over anhydrous magnesium sulfate. The residue was subjected to preparative GPC to give **uDTG** as a colorless oil in 90% yield (0.275 g, 0.578 mmol). ^1^H NMR (400 MHz, CDCl_3_) δ 7.21 (d, *J* = 2.2 Hz, 1H), 7.04 (d, *J* = 2.2 Hz, 1H), 6.70 (s, 1H), 2.52 (s, 3H), 1.47–1.41 (m, 4H), 1.33–1.20 (m, 20H), 1.17–1.10 (m, 4H), 0.87 (t, *J* = 7.0 Hz, 6H). ^13^C NMR (100 MHz, CDCl_3_) δ 146.72, 146.26, 145.83, 143.85, 141.02, 128.10, 127.97, 112.08, 32.95, 32.02, 29.37, 29.30, 25.56, 22.81, 15.65, 15.20, 14.22. HR-MS (APCI) Calcd for C_25_H_41_GeS_2_: [M + H]^+^: 479.18560, Found: 479.18591.

#### 3.2.5. Synthesis of **uDTG-Br**

A mixture of **uDTG-Si** (0.425 g, 0.683 mmol) and NBS (0.122 g, 0.683 mmol) in 15 mL of THF was stirred at 0 °C in the dark. After 4 h, the reaction was quenched with water and extracted with CH_2_Cl_2_. The organic layer and the extract were combined and washed with saturated NaCl aqueous solution (2 × 10 mL). After the organic phase was dried over anhydrous sodium sulfate and the solvent was removed under reduced pressure, the residue was purified by silica gel column chromatography eluting with hexane to give **uDTG-Br** as a yellow oil in 53% yield (0.228 g, 0.363 mmol). ^1^H NMR (400 MHz, CDCl_3_) δ 6.75 (s 1H), 2.54 (s, 3H), 1.38–1.33 (m, 4H), 1.27–1.16 (m, 24H), 0.87 (t, *J* = 7.0 Hz, 6H), 0.31 (s, 9H). ^13^C NMR (100 MHz, CDCl_3_) δ 152.76, 147.66, 146.55, 146.33, 144.37, 142.47, 127.81, 104.57, 33.00, 31.96, 29.86, 29.34, 25.58, 22.65, 16.37, 15.65, 14.25, 0.43. HR-MS (APCI) Calcd for C_28_H_48_BrGeS_2_Si: [M + H]^+^: 627.13564, Found: 629.13599.

#### 3.2.6. Synthesis of **uDTG-BTT**

uDTG-Br (14.5 mg, 0.023 mmol) and **5** (10.14 mg, 0.016 mmol) were dissolved in toluene (4 mL) that had been degassed by argon bubbling for approximately 4 h. Pd_2_(dba)_3_ (0.42 mg, 0.46 μmol) and P(*o*-tolyl)_3_ (0.56 mg, 1.84 μmol) were added to the solution, and the solution was heated to reflux with stirring for 3 days. After removal of the solvent under reduced pressure, the crude product was purified by GPC eluting with toluene to afford **uDTG-BTT** (6.7 mg, 0.048 mmol, 30%) as a dark red solid. ^1^H NMR (400 MHz, CDCl_3_) δ 8.20 (d, *J* = 3.9 Hz, 2H), 7.92 (s, 2H), 7.49 (d, *J* = 3.9 Hz, 2H), 6.71 (s, 2H), 2.46 (s, 6H), 1.44–1.37 (m, 8H), 1.32–1.11 (m, 48H), 0.87 (t, *J* = 7.0 Hz, 12H), 0.36 (s, 18H). ^13^C NMR (100 MHz, CDCl_3_) δ 152.82, 148.29, 147.54, 146.43, 146.21, 145.06, 144.26, 142.36, 139.40, 136.51, 131.70, 128.76, 128.57, 127.75, 125.91, 32.99, 31.92, 29.85, 29.34, 25.56, 22.63, 16.38, 15.66, 14.26, 0.43. HR-MS (FD) Calcd for C_70_H_100_Ge_2_N_2_S_7_Si_2_: [M]^+^: 1396.38881, Found: 1396.38577.

#### 3.2.7. Synthesis of **uDTG-BTTz**

**uDTG-BTTz** was synthesized in a similar fashion to that described for **uDTG-BTT** above, using compound **6** (29.93 mg, 0.034 mmol) instead of compound **5**. **uDTG-BTTz** was obtained as a dark red solid in 20% yield (0.95 mg, 0.68 μmol). ^1^H NMR (400 MHz, CDCl_3_) δ 8.85 (s, 2H), 8.29 (s, 2H), 6.72 (s, 2H), 2.54 (s, 6H), 1.41–1.35 (m, 8H), 1.28–1.19 (m, 48H), 0.86 (t, *J* = 7.0 Hz, 12H), 0.34 (s, 18H). ^13^C NMR (100 MHz, CDCl_3_) δ 167.09, 154.06, 152.58, 150.01, 147.48, 146.36, 146.15, 144.19, 143.35, 142.29, 137.10, 135.60, 132.79, 127.52, 122.11, 33.00, 31.96, 29.34, 29.20, 25.58, 22.86, 16.37, 15.65, 14.25, 0.44. HR-MS (FD) Calcd for C_68_H_98_Ge_2_N_4_S_7_Si_2_: [M]^+^: 1398.37930, Found: 1398.37938.

### 3.3. DFT Calculations

DFT calculations were carried out using the Gaussian 16 program [28] with the GaussView 6.0 graphical interface (Gaussian Inc., Wallingford, CT, USA), based on the B3LYP functional [29] and the 6–31G (d,p) basis set for all atoms. No solvents were involved in the calculations.

## 4. Conclusions

We have synthesized unsymmetrically condensed benzothienogermole **BTG** and dithienogermole **uDTG** and thoroughly investigated their optical and electrochemical properties. DFT calculations have also been carried out. Replacing the benzene ring in **BTG** with a thiophene ring changes the electronic states to enhance conjugation in uDTG derivatives. We have also explored the potential application of **uDTG** as a donor unit in conjugated D–A materials by synthesizing D–A compounds **uDTG-BTT** and **uDTG-BTTz** featuring the combination with a benzothiadiazole acceptor unit. **uDTG-BTT** and **uDTG-BTTz** exhibited extended conjugation and clear solvatochromic behavior arising from their intramolecular D–A interaction. These results indicate the potential application of the dithieno[3,2-*b*:4,5-*c*’]germole unit as an electron donor in D–A systems. Studies on the preparation of D–A materials based on the uDTG system including conjugated polymers are in progress, the results of which will be reported elsewhere.

## Figures and Tables

**Table 1:** Optical and electrochemical properties of triazologermoles.

Compound	UV abs. λ_max_^a^/nm	PL λ_max_^a,b^/nm (Φ_f_)	HOMO ^c^/eV	LUMO ^d^/eV	LUMO–HOMO ^e^/eV
**BTG**	280	323 (<2%)	−6.27	−2.31	3.96
**uDTG**	303	347 (3.8%)	−5.50	−1.73	3.77
**uDTG-Si**	315	360 (<2%)	−5.52	−1.90	3.62
**uDTG-BTT**	329, 520	653 (33%)	−5.08	−3.04	2.04
**uDTG-BTTz**	331, 501	634 (41%)	−5.16	−3.05	2.11

^a^ In dichloromethane at room temperature. ^b^ Excited at the absorption maximum. ^c^ Derived from CV anodic onset potential. ^d^ E (LUMO) = E (HOMO) + *Eg*. ^e^ Optical band gap (*Eg*).

## Data Availability

Data are contained within the article and Supplementary Materials.

## Supplementary Materials

The following supporting information can be downloaded at https://www.mdpi.com/article/10.3390/molecules29153553/s1. Figure S1: UV absorption (solid line) and PL (dotted line) spectra (**a**) and the Lippert–Mataga plot (**b**) of **uDTG-BTTz.** The slope of the fitted line is 5405 cm^−1^ (R^2^ = 0.87); Figure S2: Photograph of **uDTG-BTT** in several solvents under 365 nm at room temperature; Table S1: Absorption maxima, emission maxima, and Stokes’ shift of **uDTG-BTT** and **uDTG-BTTz** in various solvents; Figure S3: Cyclic voltammograms of **BTG**, **uDTG**, **uDTG-Si**, **uDTG-BTT**, and **uDTG-BTTz** in DCM/TBAHFP (0.1 M), [c] = 1 × 10^−4^ mol L^−1^, 298 K, scan rate = 50 mV s^−1^, and 5; and Figures S4–S17: NMR spectra of newly prepared compounds in the present study. Reference [30] is cited in the Supplementary Materials.
