# Supplementary material for: The Preparation of Dithieno[3,2-b:4,5-c’]germole, and Its Application as a Donor Unit in Conjugated D–A Compounds
Source: Molecules. 2024 Jul 28;29(15):3553. doi: 10.3390/molecules29153553 (PMC11313974; doi:10.3390/molecules29153553)
Supplement: Supplementary file 1 [file molecules-29-03553-s001.zip › molecules-3117847-supplementary.pdf]

Supporting Information

Preparation of Dithieno[3,2-*b*:4,5-*c*']germole and Its Application as a Donor Unit in Conjugated D–A Oligomers

Cong-Huan Wang,[1] Yohei Adachi,[1] Joji Ohshita[1,2,*]

[1] Smart Innovation Program, Graduate School of Advanced Science and Engineering, Hiroshima University, Higashi-Hiroshima 739-8527, Japan

[2] Division of Materials Model-Based Research, Digital Monozukuri (Manufacturing) Education and Research Center, Hiroshima University, Higashi-Hiroshima 739-0046, Japan

Content

1. **Figure S1.** UV absorption (solid line) and PL (dotted line) spectra (a) and the Lippert-Mataga plot (b) of **uDTG-BTTz**. The slope of the fitted line is 5405 cm–1 (R2 = 0.87) . →S2
2. **Figure S2.** Photograph of **uDTG-BTT** in several solvents under 365 nm at room temperature. →S2
3. **Table S1.** Absorption maxima, emission maxima, and Stokes' shift of **uDTG-BTT** and **uDTG-BTTz** in various solvents. →S2
4. **Figure S3.** Cyclic voltammograms of **BTG**, **uDTG**, **uDTG-Si**, **uDTG-BTT**, and **uDTG-BTTz** in DCM/TBAHFP (0.1 M), [c] = 1 × 10^{-4} mol L^{-1}, 298 K, scan rate = 50 mV s^{-1}. →S3
5. **Figures S4-S17.** NMR spectra of newly prepared compounds in the present study. →S4-10

(a)

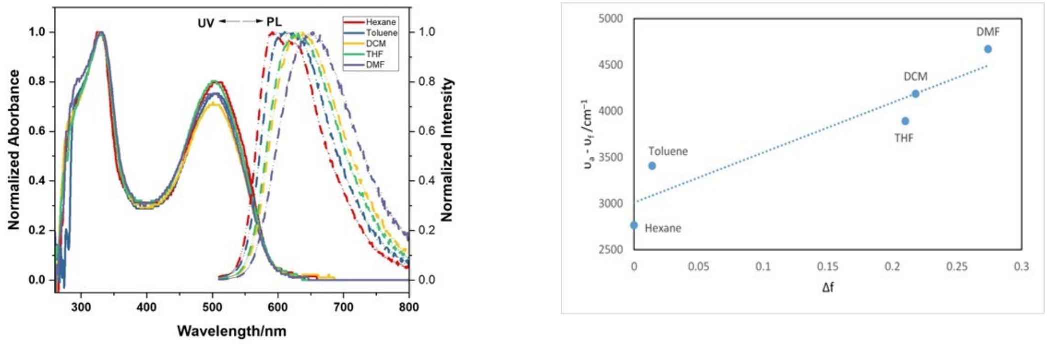

Figure S1. UV absorption (solid line) and PL (dotted line) spectra (a) and the Lippert-Mataga plot (b) of **uDTG-BTTz**. The slope of the fitted line is 5405 cm–1 (R2 = 0.87).

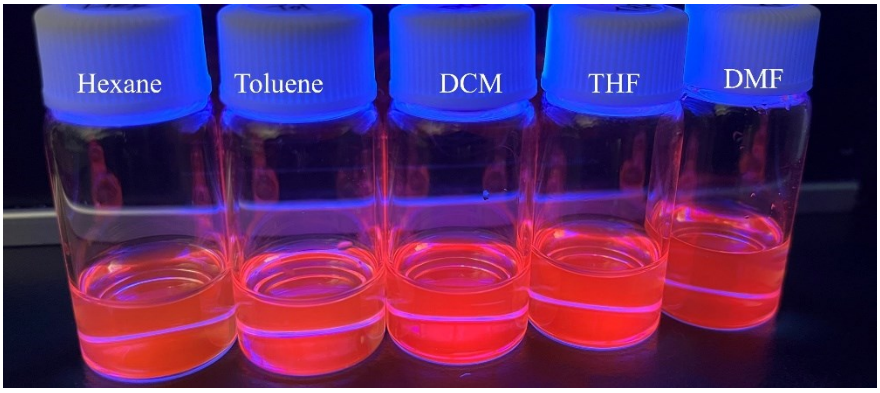

Figure S2. Photograph of **uDTG-BTT** in several solvents under 365 nm at room temperature.

Table S1. Absorption maxima, emission maxima, and Stokes' shift of **uDTG-BTT** and **uDTG-BTTz** in various solvents

Solvents	Δf	**uDTG-BTT**			**uDTG-BTTz**		
		λ_a /nm	λ_f /nm	Stokes Shift/cm^{-1}	λ_a /nm	λ_f /nm	Stokes Shift/cm^{-1}
Hexane	0	526	611	2644	508	591	2764
Toluene	0.014	525	625	3047	505	610	3408
CH_2Cl_2	0.218	521	647	3737	501	634	4187
THF	0.210	524	647	3628	504	627	3892
DMF	0.309	523	662	4014	501	654	4670

The Lippert-Mataga equation [(S1), (S2)]

$$v_a - v_f = \frac{2(\mu_e - \mu_g)^2}{hc\alpha^3} \Delta f + \text{const} \qquad (S1)$$

$$\Delta f = \left[\frac{\varepsilon - 1}{2\varepsilon + 1}\right] - \left[\frac{n^2 - 1}{2n^2 + 1}\right] \qquad (S2)$$

where ν_a and ν_f are the absorption and fluorescence band maximum positions (cm^{-1}),

respectively, ε is the dielectric constant of the solvent medium, and n is the refractive index of the medium. The quantities h, c, a, μg and μe are Planck's constant, the velocity of light, the Onsager cavity radius, the ground and excited state dipole moments, respectively. The detailed parameters of the solvents were taken from references [30].

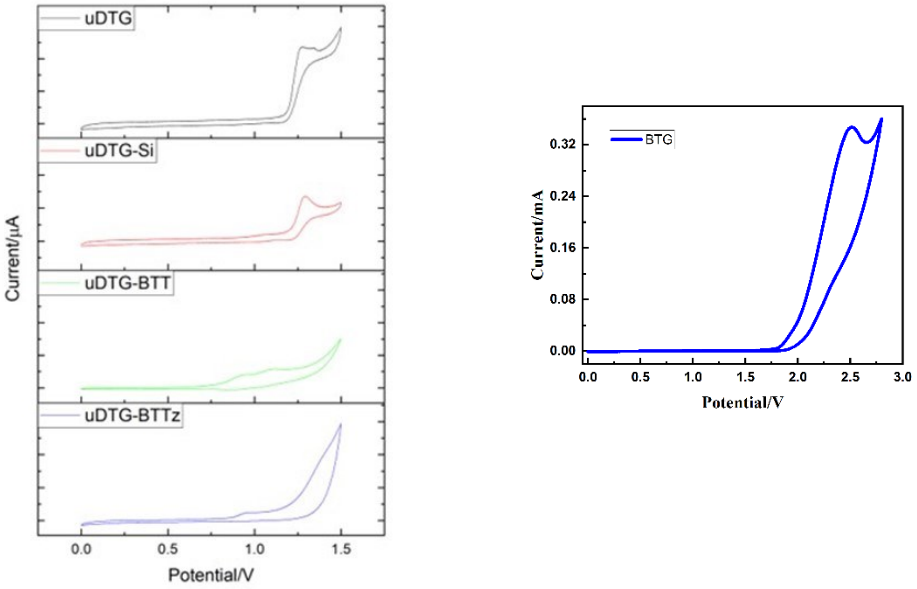

Figure S3. Cyclic voltammograms of **BTG**, **uDTG**, **uDTG-Si**, **uDTG-BTT**, and **uDTG-BTTz** in DCM/TBAHFP (0.1 M), [c] = 1 × 10^{-4} mol L^{-1}, 298 K, scan rate = 50 mV s^{-1}.

NMR spectra

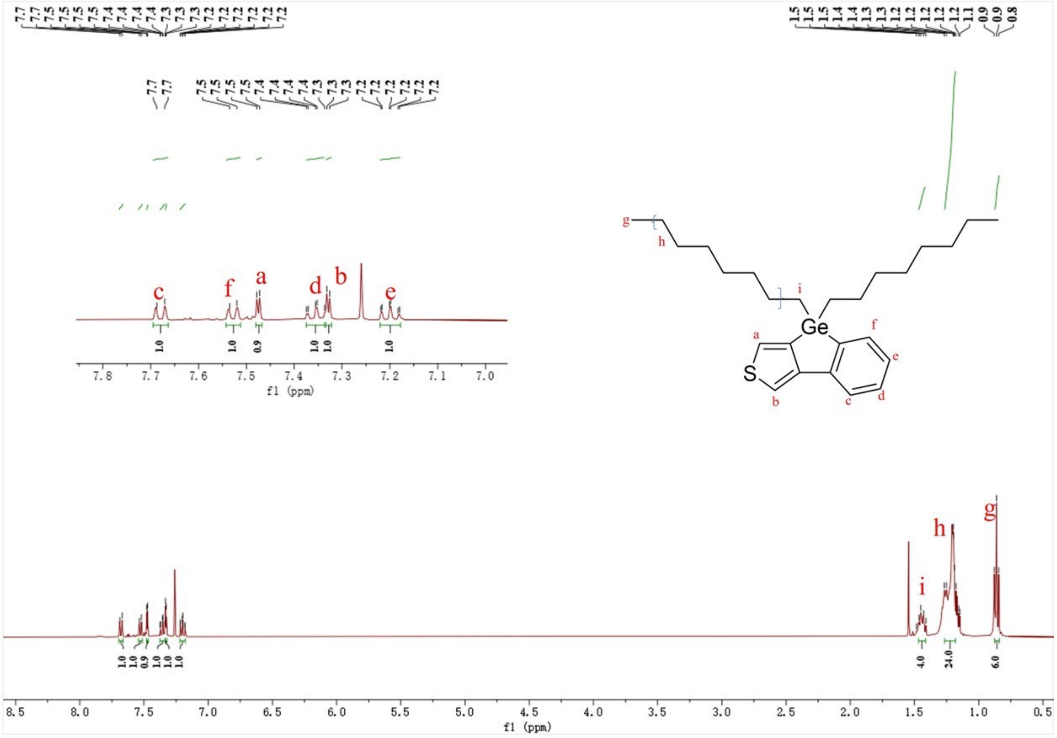

Figure S4. ^{1}H NMR spectrum of **BTG** in $CDCl_3$.

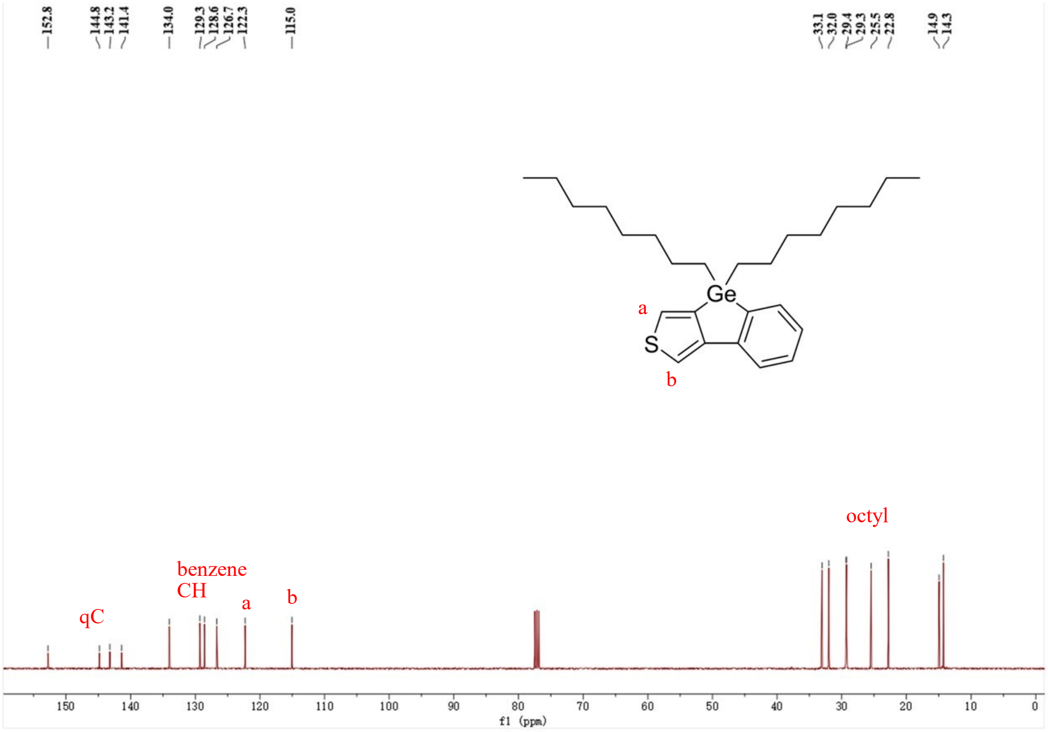

Figure S5. ^{13}C NMR spectrum of **BTG** in $CDCl_3$.

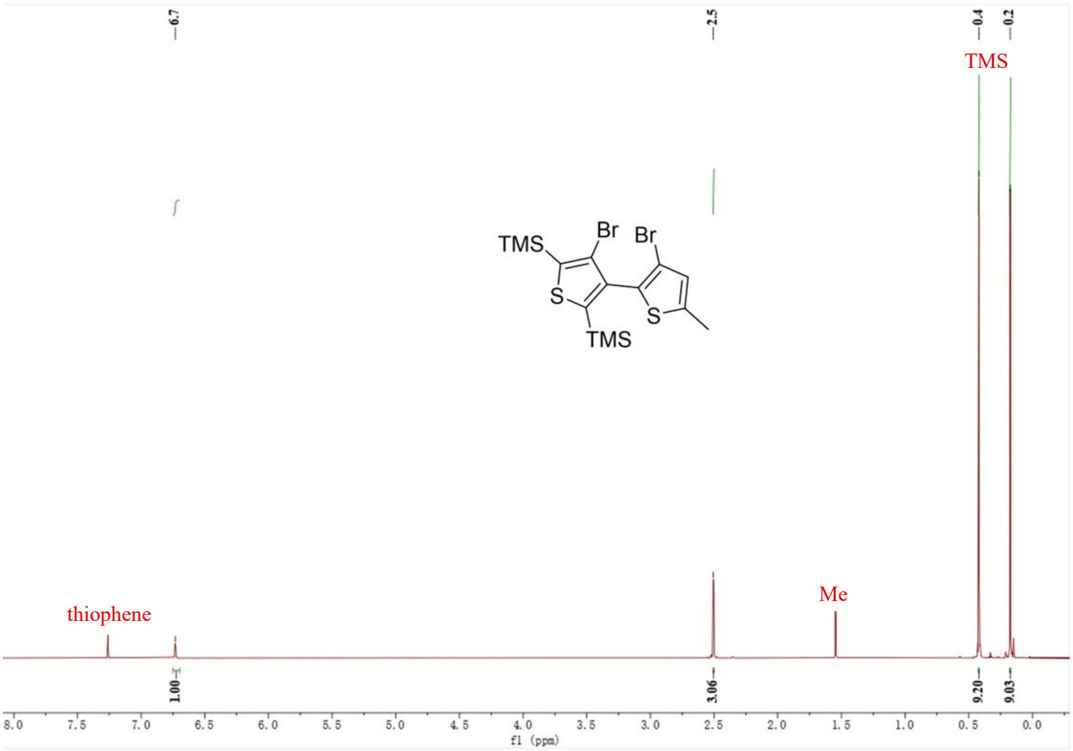

Figure S6. ^{1}H NMR spectrum of **4** in $CDCl_3$.

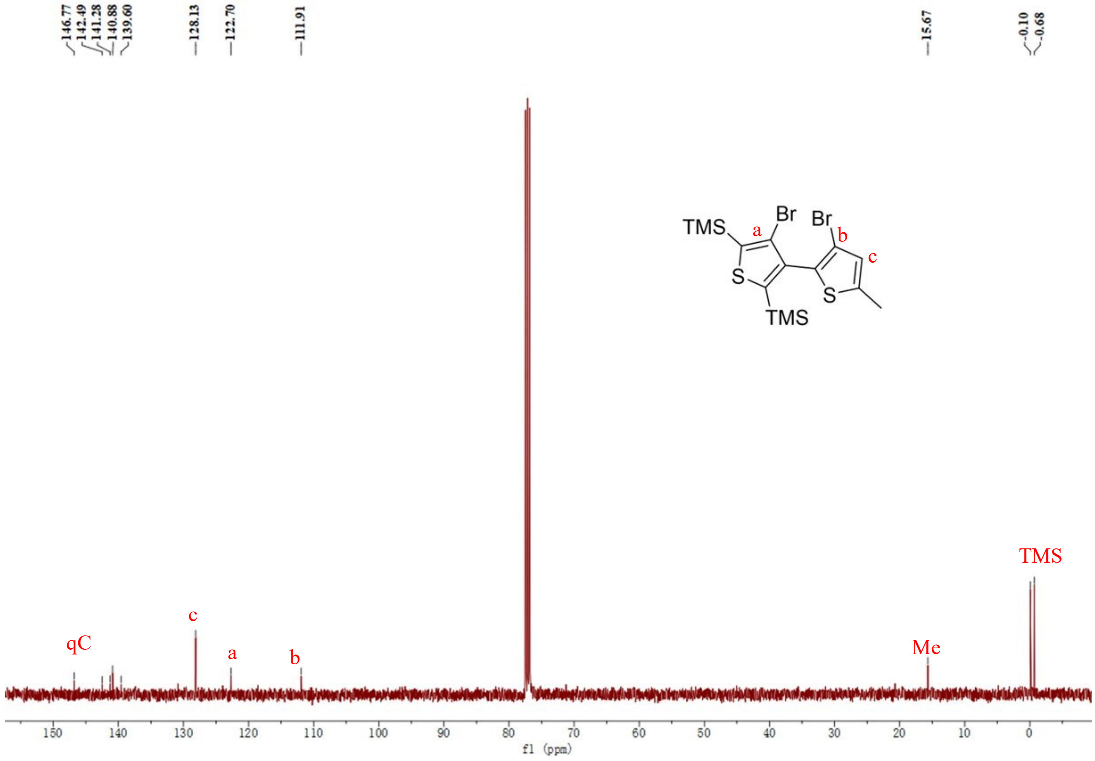

Figure S7. ^{13}C NMR spectrum of **4** in $CDCl_3$.

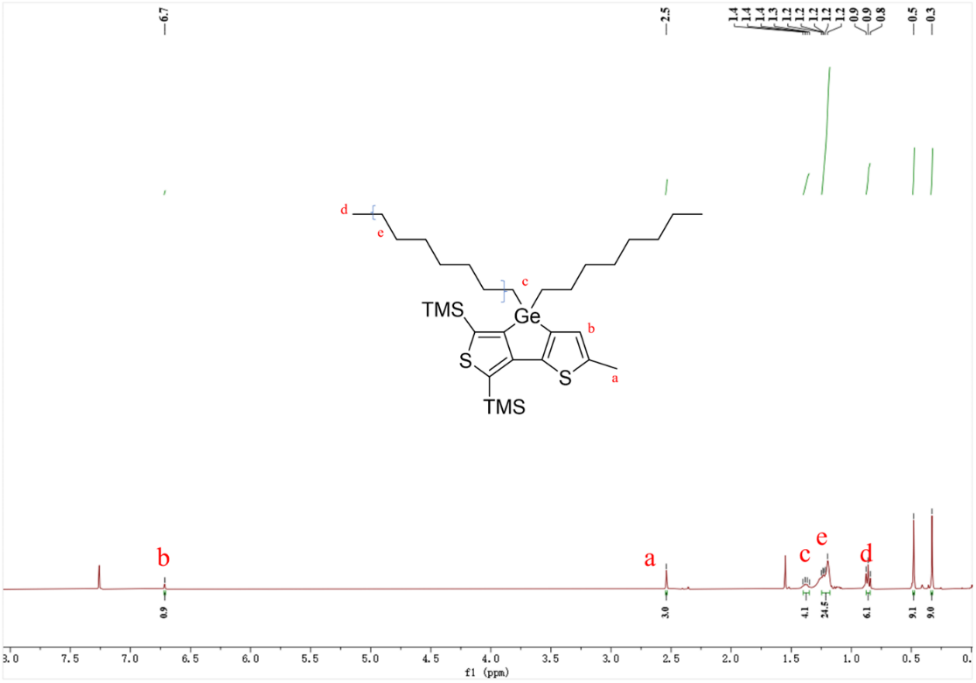

Figure S8. ^{1}H NMR spectrum of **uDTG-Si** in $CDCl_3$.

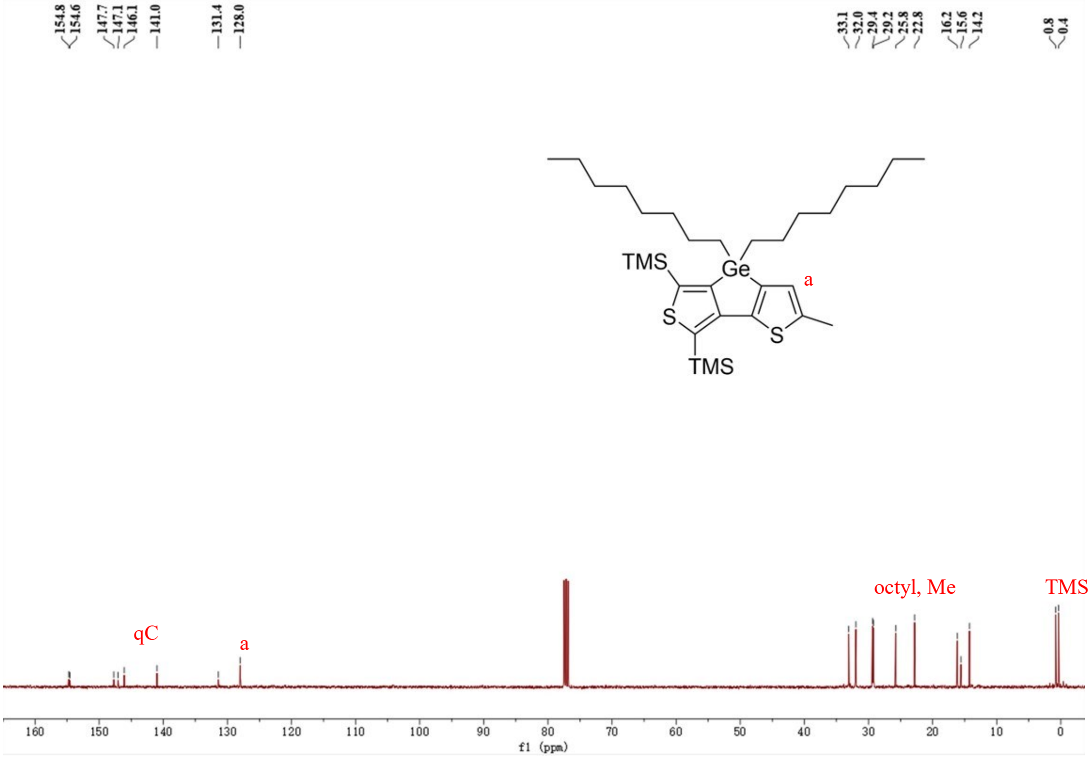

Figure S9. ^{13}C NMR spectrum of **uDTG-Si** in $CDCl_3$.

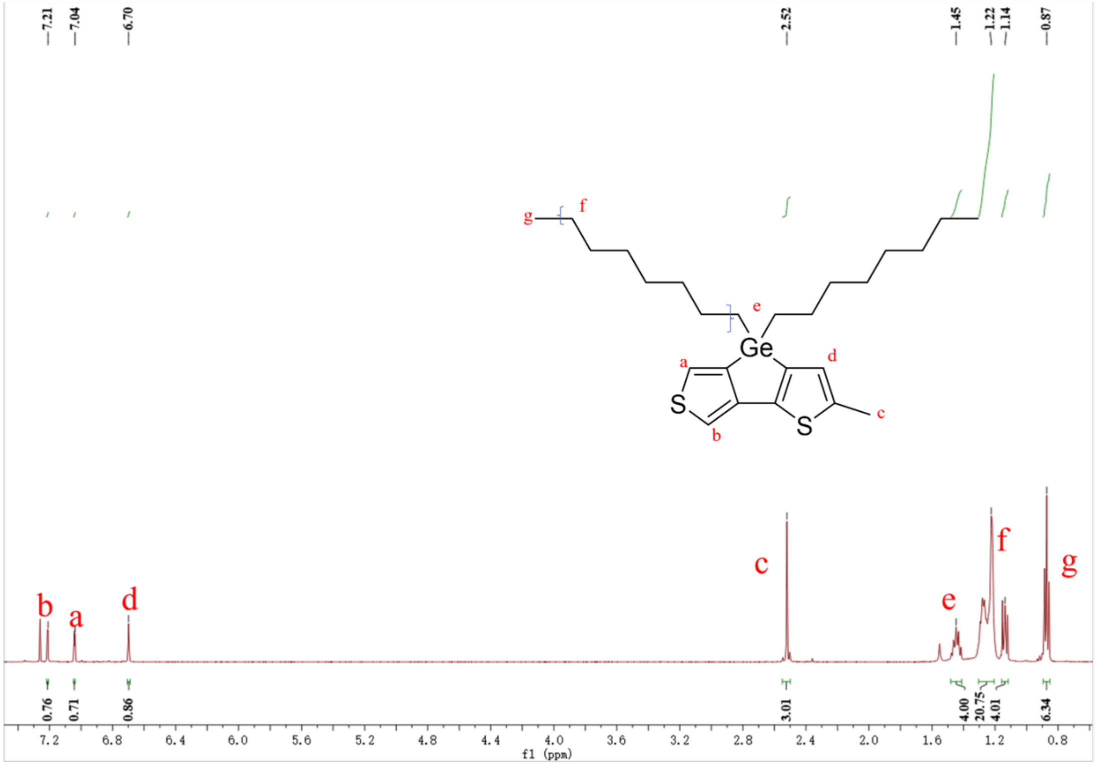

Figure S10. ^{1}H NMR spectrum of **uDTG** in $CDCl_3$.

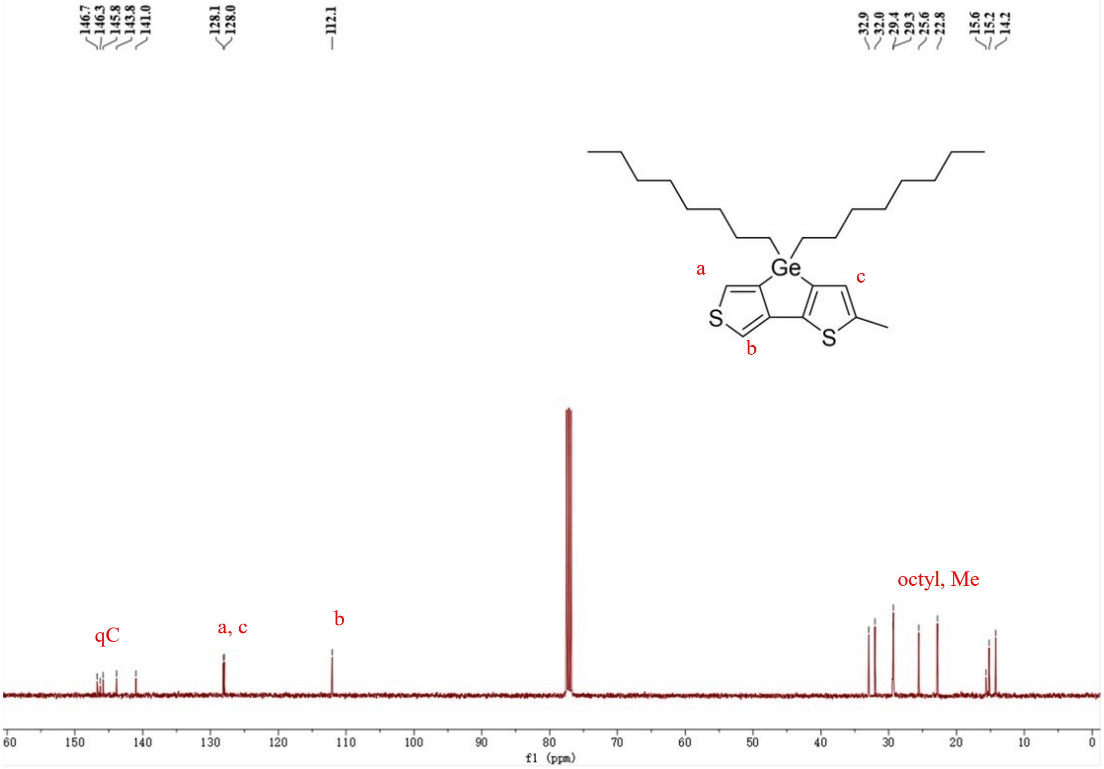

Figure S11. ^{13}C NMR spectrum of **uDTG** in $CDCl_3$.

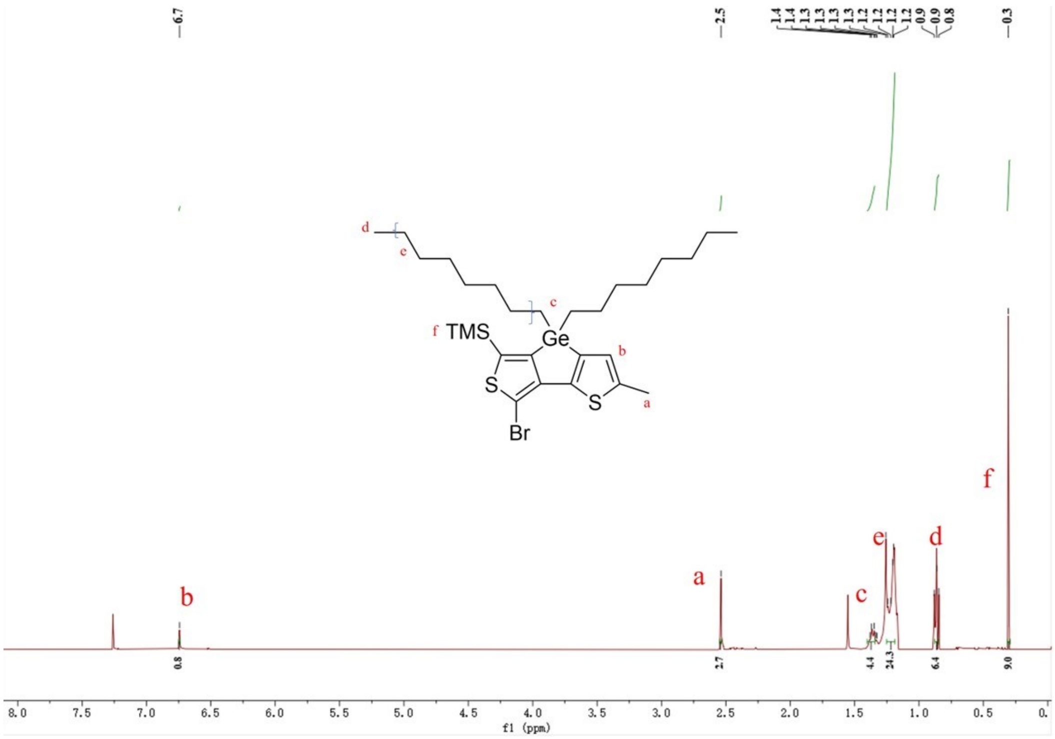

Figure S12. ^{1}H NMR spectrum of **uDTG-Br** in $CDCl_3$.

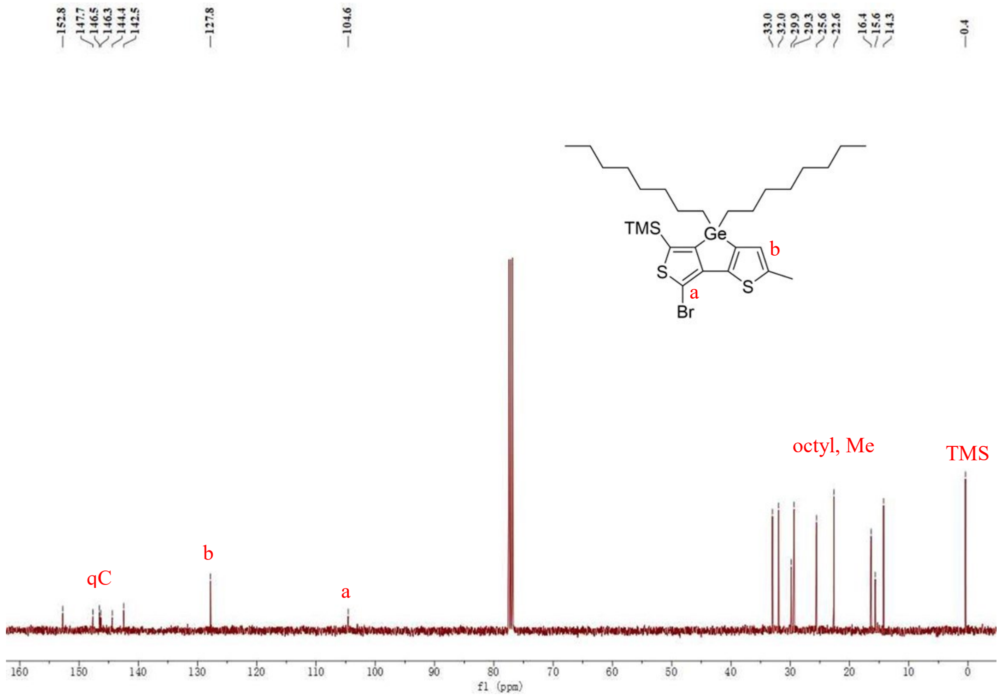

Figure S13. ^{13}C NMR spectrum of **uDTG-Br** in $CDCl_3$.

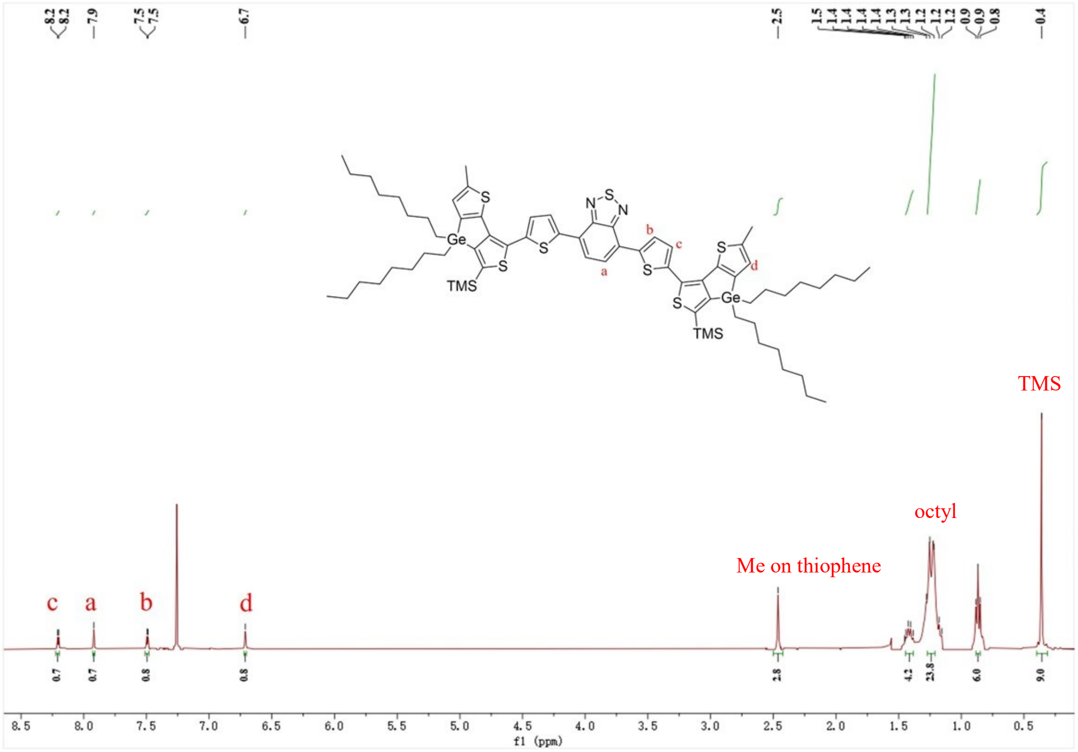

Figure S14. ^{1}H NMR spectrum of **uDTG-BTT** in $CDCl_3$.

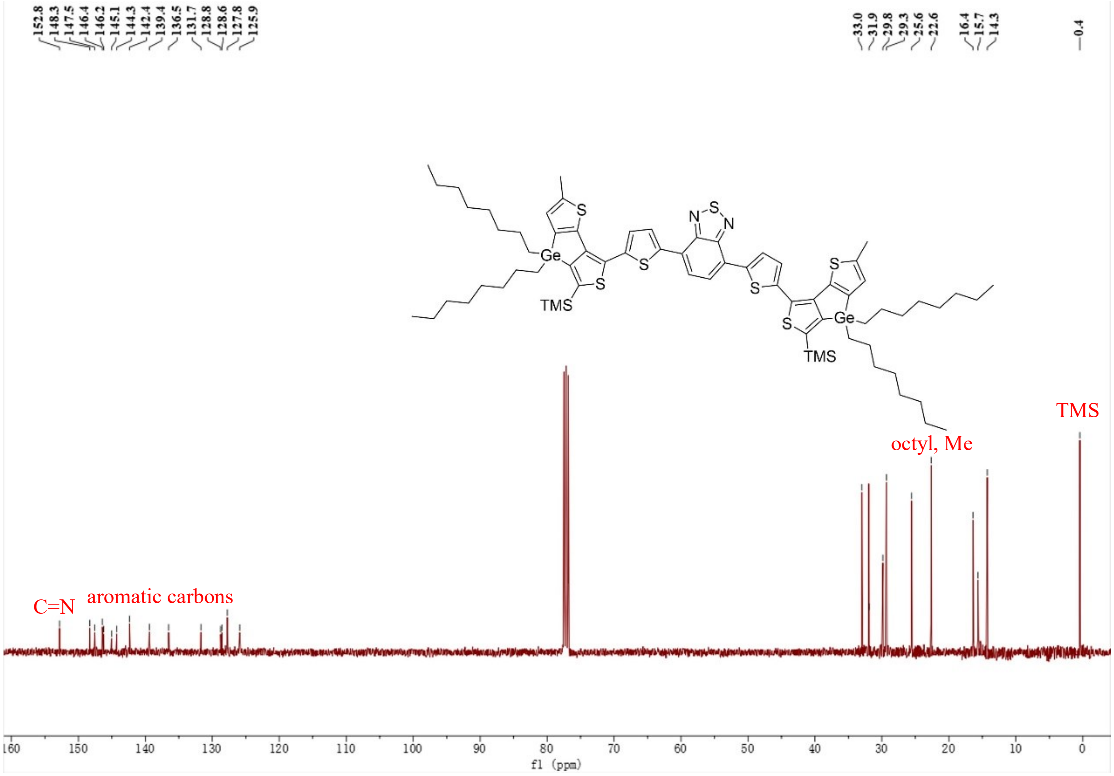

Figure S15. ^{13}C NMR spectrum of **uDTG-BTT** in $CDCl_3$.

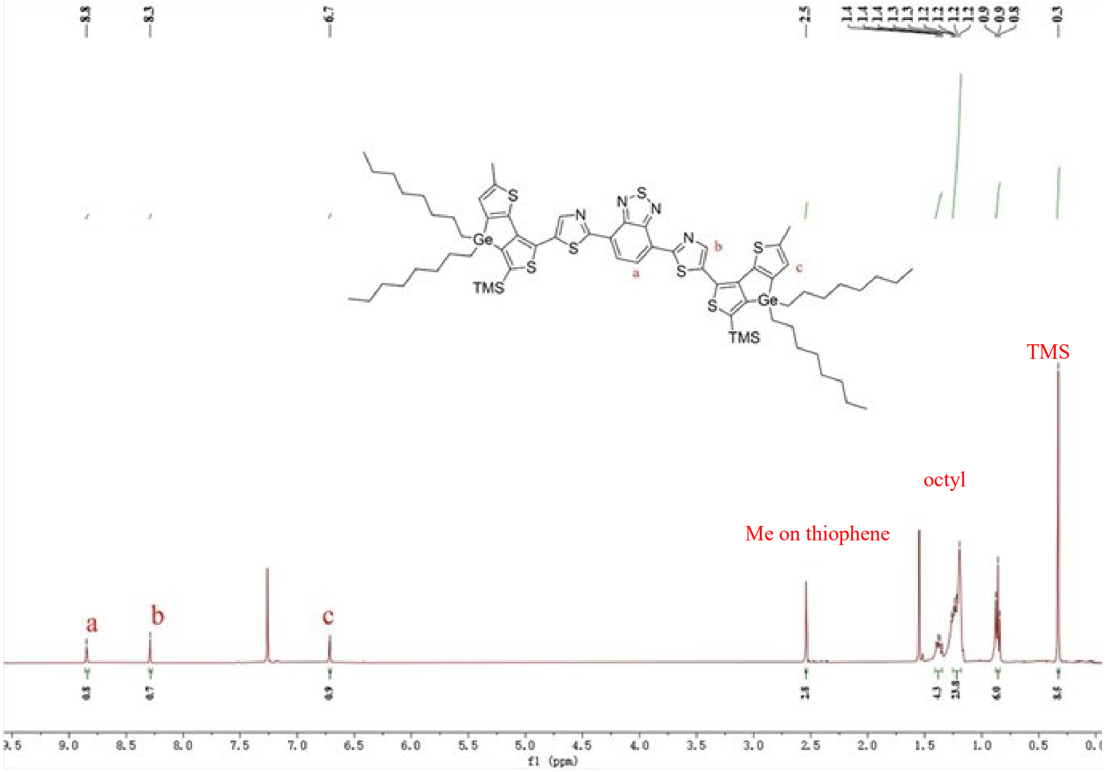

Figure S16. ^{1}H NMR spectrum of **uDTG-BTTz** in $CDCl_3$

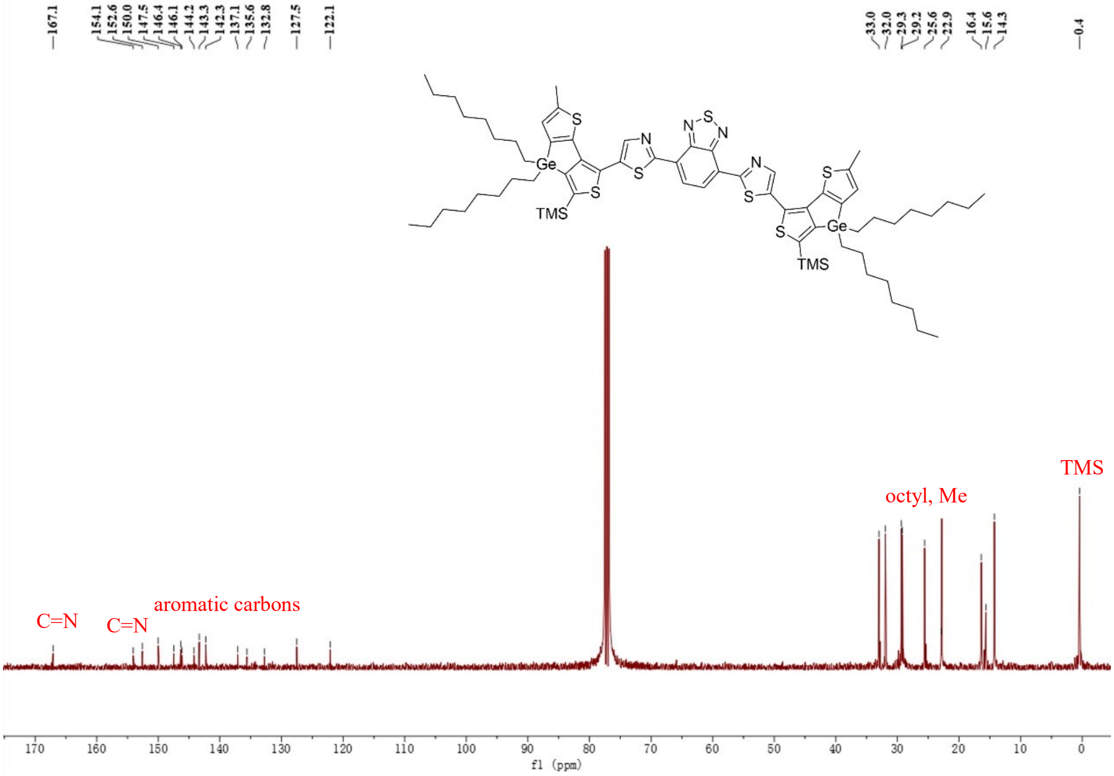

Figure S17. ^{13}C NMR spectrum of **uDTG-BTTz** in $CDCl_3$.

Reference

[30] Pandey, N.; Tewari, N.; Pant, S.; Mehata, M. S. Solvatochromism and Estimation of Ground and Excited State Dipole Moments of 6-Aminoquinoline. *Spectrochim Acta A Mol Biomol Spectrosc.* **2022**, *267*, 120498.
